# Dataset of material measurement based on SEM images of Ag/TiO_2_ nanocomposite material synthesized via Horizontal Vapor Phase Growth (HVPG) technique

**DOI:** 10.1016/j.dib.2019.105018

**Published:** 2020-01-03

**Authors:** Muhammad Akhsin Muflikhun, Alvin Y. Chua, Gil Nonato C. Santos

**Affiliations:** a Department of Mechanical and Industrial Engineering, Gadjah Mada University, Indonesia; b Mechanical Engineering Department, De La Salle University, Manila, Philippines; c Physics Department, De La Salle University, Manila, Philippines

**Keywords:** HVPG technique, Ag/TiO_2_, Synthesis nanocomposite, SEM, Material measurement

## Abstract

This data describes about the measurement technique of Ag/TiO_2_ nanocomposite materials that successfully synthesized via Horizontal Vapor Phase Growth (HVPG) technique. The data are obtained after specimens were placed in the Scanning Electron Microscope (SEM) chamber to be analyzed. The present data were captured from SEM with different magnification. There are total 27 variable data to be analyzed from three different parameters; growth temperature, baking time and zones. In total, 9 different quartz tubes that contains of Ag/TiO_2_ nanocomposite material are evaluated. Data are described in average value where the different calculations are presented. Raw data are also embedded in the Appendix for further analysis purposes. These data can be useful as the information of size measurement of Ag/TiO_2_ nanocomposite materials in different temperature and time during synthesis process.

**Table:** Specifications Table

Subject	Engineering
Specific subject area	Material science and engineering, Nanotechnology
Type of data	Table Figure
How data were acquired	SEM JEOL JSM-5310 with SemAfore software
Data format	Raw and Analyzed
Parameters for data collection	Shape and diameter measurement of nanocomposite material were harvested from SEM images of Ag/TiO_2_ nanocomposite materials synthesized by using HVPG technique. This technique used 2 parameters during fabrication process; Growth temperature and Baking time.
Description of data collection	The present data are based on 27 different zones that based on 3 different growth temperatures, 3 different baking times, and in each sample was divided into 3 different zones based on quartz tube locations.
Data source location	Solid state physics lab, Dela Salle University, Manila, Philippines
Data accessibility	Data provided in the article are accessible to the public.

**Table:** 

**Value of the Data** • The data are valuable for understanding how to measure the nanocomposite material by using SEM analysis. • The data are useful to predict the size of Ag/TiO2 nanocomposite materials in different combinations. • The data can be used for clustering process of the shape of nanocomposite materials. Clustering the shape of material can help engineer, scientists, and designer to use different shape with different size for different purposes. • The data provide how measurement are done and this can be easy to replicate by other researchers.

## 1 Data description

**Fig. 1:**
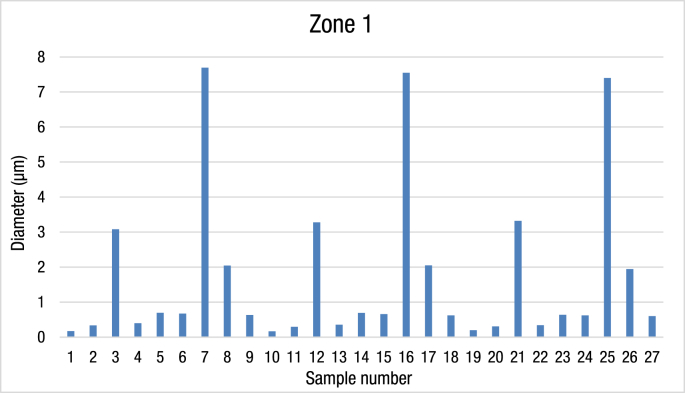
The average diameter of Ag/TiO_2_ nanocomposite material in zone 1.

**Fig. 2:**
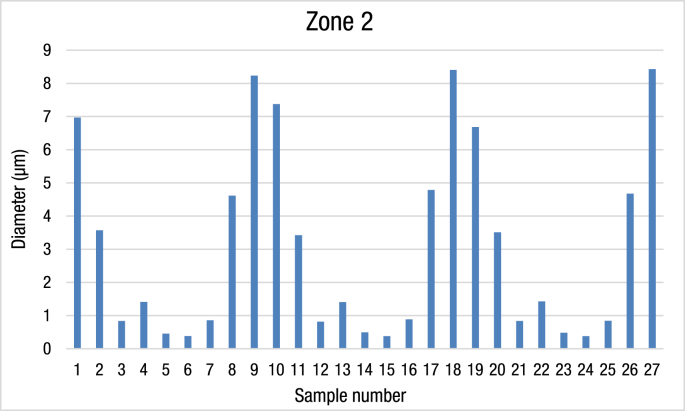
The average diameter of Ag/TiO2 nanocomposite material in zone 2.

**Fig. 3:**
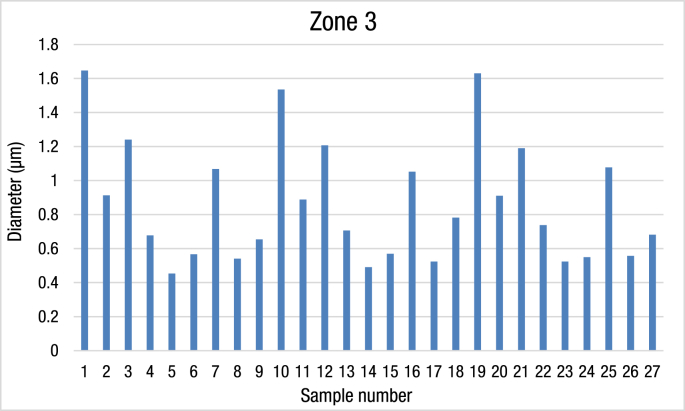
The average diameter of Ag/TiO2 nanocomposite material in zone 3.

The data consist of material diameter of Ag and TiO_2_ raw material, and Ag/TiO_2_ nanocomposite material. A total of 27 different zones was measured with at least three different locations were used as data sampling. SEM image analysis was used to measure the diameter of the materials. For instance, representative figures are shown with the measurement method. Table 1 and Fig. 1 show the average diameter measurement of Ag/TiO_2_ nanocomposite material in zone 1. The measurement technique can be divided into three different baking times, and three different growth temperatures, the tube also divided into three zones and can be obtained as well. Table 2 and Fig. 2 presented data on the diameter measurement of Ag/TiO_2_ in zone 2. The data are obtained based on average measurement after conducted in 3 different measurements. Table 3 and Fig. 3 revealed the average measurement of Ag/TiO_2_ nanocomposite materials in zone 3. The raw data of SEM images from 27 zones within 9 different tubes are shown in supplementary file of this paper.

**Table 1:** The average of Ag/TiO_2_ nanocomposite diameter from zone 1.

Zone 1				
No.	Temp. (°C)	Baking Time (h)	Zone	Diameter (μm)
1	800	4	1	0.171333
2	800	6	1	0.333
3	800	8	1	3.08
4	1000	4	1	0.399667
5	1000	6	1	0.694667
6	1000	8	1	0.672
7	1200	4	1	7.693333
8	1200	6	1	2.043333
9	1200	8	1	0.632
10	800	4	1	0.162
11	800	6	1	0.298667
12	800	8	1	3.276667
13	1000	4	1	0.354
14	1000	6	1	0.691
15	1000	8	1	0.659
16	1200	4	1	7.546667
17	1200	6	1	2.05
18	1200	8	1	0.619
19	800	4	1	0.196333
20	800	6	1	0.307333
21	800	8	1	3.32
22	1000	4	1	0.339667
23	1000	6	1	0.639667
24	1000	8	1	0.623
25	1200	4	1	7.4
26	1200	6	1	1.943333
27	1200	8	1	0.599

**Table 2:** The average of Ag/TiO2 nanocomposite diameter from zone 2.

Zone 2				
No.	Temp. (°C)	Baking Time (h)	Zone	Diameter (μm)
1	800	4	2	6.97
2	800	6	2	3.57333
3	800	8	2	0.839
4	1000	4	2	1.41333
5	1000	6	2	0.45533
6	1000	8	2	0.38467
7	1200	4	2	0.861
8	1200	6	2	4.61333
9	1200	8	2	8.23
10	800	4	2	7.37333
11	800	6	2	3.42
12	800	8	2	0.81767
13	1000	4	2	1.40667
14	1000	6	2	0.49333
15	1000	8	2	0.382
16	1200	4	2	0.886
17	1200	6	2	4.78667
18	1200	8	2	8.40333
19	800	4	2	6.68667
20	800	6	2	3.51
21	800	8	2	0.84033
22	1000	4	2	1.42667
23	1000	6	2	0.483
24	1000	8	2	0.38033
25	1200	4	2	0.84333
26	1200	6	2	4.67333
27	1200	8	2	8.43

**Table 3:** Table 2 the average of Ag/TiO2 nanocomposite diameter from zone 3.

Zone 3				
No.	Temp. (°C)	Baking Time (h)	Zone	Diameter (μm)
1	800	4	3	1.64667
2	800	6	3	0.913
3	800	8	3	1.24
4	1000	4	3	0.677
5	1000	6	3	0.45333
6	1000	8	3	0.567
7	1200	4	3	1.06767
8	1200	6	3	0.541
9	1200	8	3	0.654
10	800	4	3	1.53567
11	800	6	3	0.88867
12	800	8	3	1.20667
13	1000	4	3	0.70667
14	1000	6	3	0.491
15	1000	8	3	0.56933
16	1200	4	3	1.052
17	1200	6	3	0.52433
18	1200	8	3	0.78233
19	800	4	3	1.63
20	800	6	3	0.91033
21	800	8	3	1.19
22	1000	4	3	0.73767
23	1000	6	3	0.52433
24	1000	8	3	0.54933
25	1200	4	3	1.07767
26	1200	6	3	0.557
27	1200	8	3	0.681

Detail measurements of all parameters can be seen in Table 4 included source material (raw material of Ag and TiO_2_ powders). The source data also provided in the appendix.

**Table 4:**
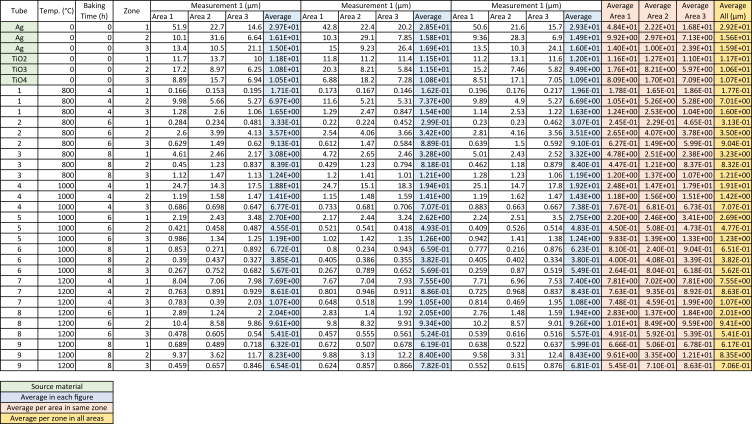
All measurement of Ag/TiO_2_ nanocomposite materials.

## 2 Experimental design, materials, and methods

### 2.1 Design, materials, and methods

There are many methods to synthesis various materials with many combinations [1], included synthesis silver and titanium dioxide nanocomposite materials [2]. El-Nour et al. [3] describe there are 2 major synthesis of Ag nanoparticle, which are physical approach and chemical approach. Krutyakov et al. [4] also explained the synthesis Ag nanoparticles that divided into two synthesis methods, which are conventional and unconventional methods. Natsuki et al. [5] explained the synthesis Ag nanoparticle using 4 main methods, which are Physical method, photochemical method, Biological method, and chemical method. A more detailed explanation about synthesis TiO_2_ are proposed by Chen and Mao [6], where they explained that there are 11 main methods that can be used to synthesis TiO_2_ nanomaterial such as; Sol-gel method, Michelle and Inverse Michelle method, Sol method, hydrothermal method, Solvothermal method, Direct oxidation method, Chemical vapor deposition method, Physical vapor deposition method, and Electrodeposition method, Microwave method, and Sonochemical method.

Among many methods that can be used to synthesize Ag and TiO_2_, Physical Vapor Deposition (PVD) method is one of the simplest methods with high purity output. The materials are evaporated in the high temperature and reach its melting point. The material then thermally deposited in the lower temperature and then condensed to form as a solid. High temperature and low pressure are used to increase the synthesis process.

**Fig. 4:**
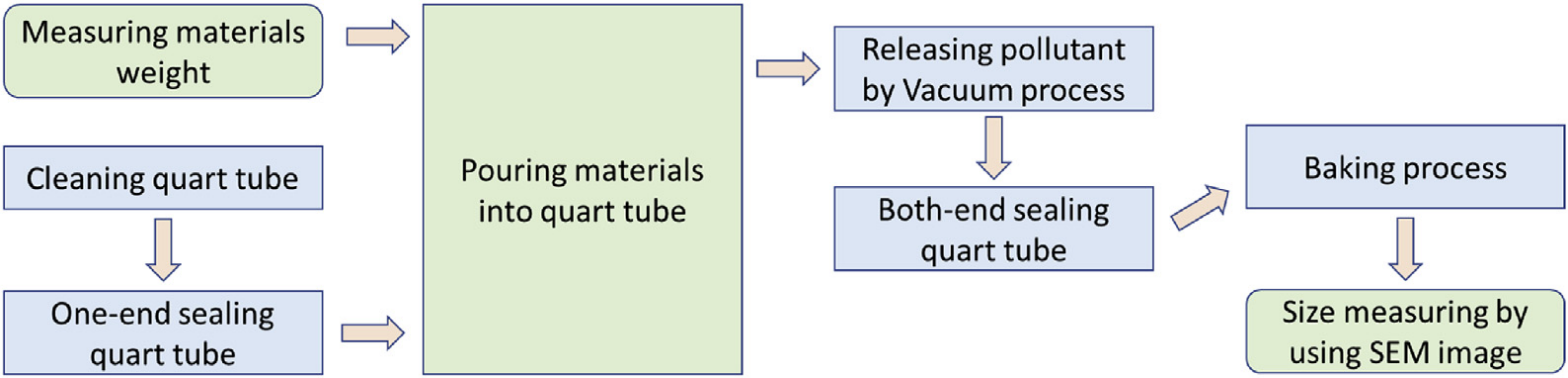
HVPG technique flowchart to synthesis Ag/TiO_2_ nanocomposite materials.

**Fig. 5:**
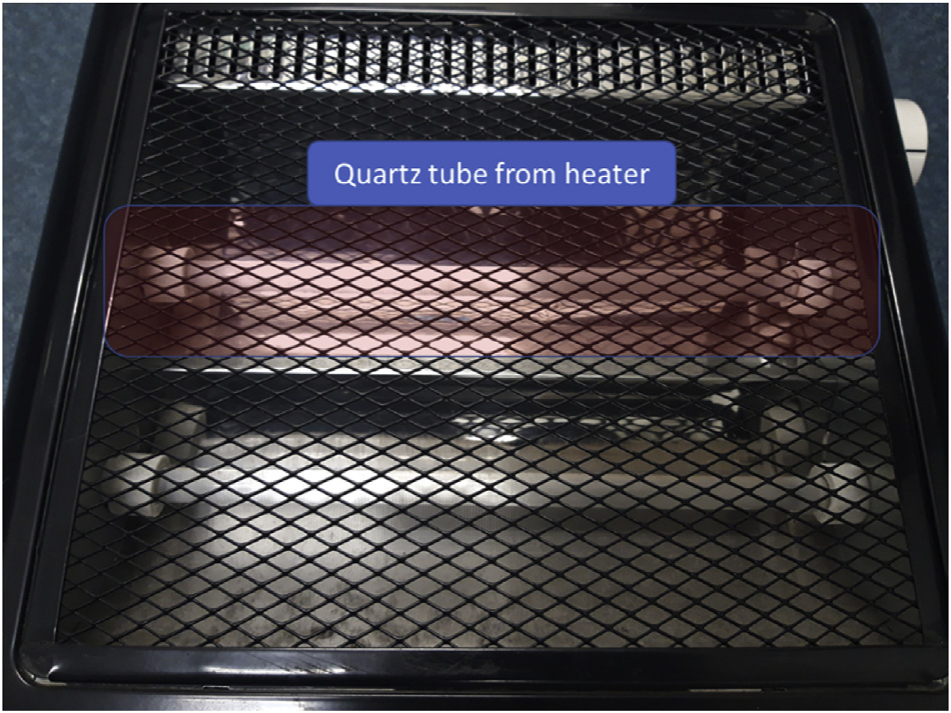
Quartz tube used for synthesis Ag/TiO_2_ nanocomposite material.

The present paper used a modified method of PVD to reduce the cost by using quart tube. Since the process use quartz tube and it were placed in the furnace with horizontal position, the method is called Horizontal Vapor Phase Growth (HVPG) technique. The sequence of synthesis Ag/TiO_2_ nanocomposite material can be described in Fig. 4. Detail method with a detailed flowchart of HVPG technique can be found in Muflikhun et al. [7]. Source material consists of 17.5 mg Ag powder from Aldrich Corporation and 17.5 mg TiO_2_ from Degussa P25 were prepared. As a medium for growth nanocomposite, cheap quartz tube from heater components was used as shown in Fig. 5. The tube then washed and cleaned before sealing with a blowtorch at one-end. Before materials poured into the tube, the powder that contains of Ag and TiO_2_ were mixed to gain equal distribution in each part.

The tube that contains material (powder) then placed in the vacuum machine to remove air and pollutant that may occur. After the condition inside the tube becomes a vacuum with pressure reach 10^−6^ Torr, the tube then sealed in the both-end. The tubes, then placed vertically in the furnace with half position inside the furnace and half position outside the furnace. Temperature of growth and baking time is set from the furnace with automatic off after the time fulfilled.

After cooling process, the tubes that contain nanocomposite materials then cracked by using a gavel with a slow push to avoid material inside the tube to move. Small sample in each zone then placed in the SEM sample plate to be analyzed.

### 2.2 Measurement technique

Since it was invented by German researcher and inventor, Manfred von Ardenne in 1938, SEM is used by many scientists all around the world for analyzing and constructing tiny object to nanoscale materials. Over the past decades, development of SEM to attain higher precision, clearer image output, and faster operation process have made the nanotechnology field and analysis nanomaterial more attractive for researchers [8].

The main function of SEM is like a microscope in general, to see small objects to be seen clearer. The main difference with light microscope is the scope of SEM can have magnification to look the object up to nano scale. This ability makes SEM is used by many researchers as the vital equipment to develop new material at nano level [9].

The data shows SEM image was capable to analysis the size and measure the diameter and size of nanocomposite material that consist of Ag/TiO_2_. Since Ag/TiO_2_ nanocomposite material was reported by many researchers that can be used in various applications, for instance: anti-bacterial application [10], clean and renewable fuel [11], sensor application [12], and multifunctional applications (UV resistance, UV protection, and increase wear properties) [13].

**Fig. 6:**
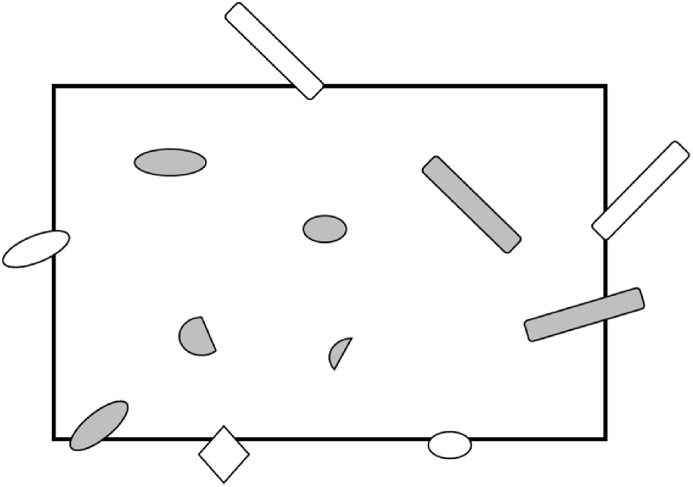
Treatment of objects that cut by the edge from SEM image.

The material measurement used random object that clearly placed in one frame. Furthermore, the measurement technique used ISO 13322-1 as a standard for measuring object [14]. The parts choice based on the object that fully located in the scope of one frame. The object that out of frame is excluded except the main body of the object can be fully recognized. At least 3 measurements are done in one frame. The object that measured are allocated in the frame with all parts of the object is shown clearly and all objects to be measured is within the frame scope. The object analysis is illustrated in Fig. 6. Grey objects can be measured and calculated, and the white object is expelled from measured and not be calculated.

**Fig. 7:**
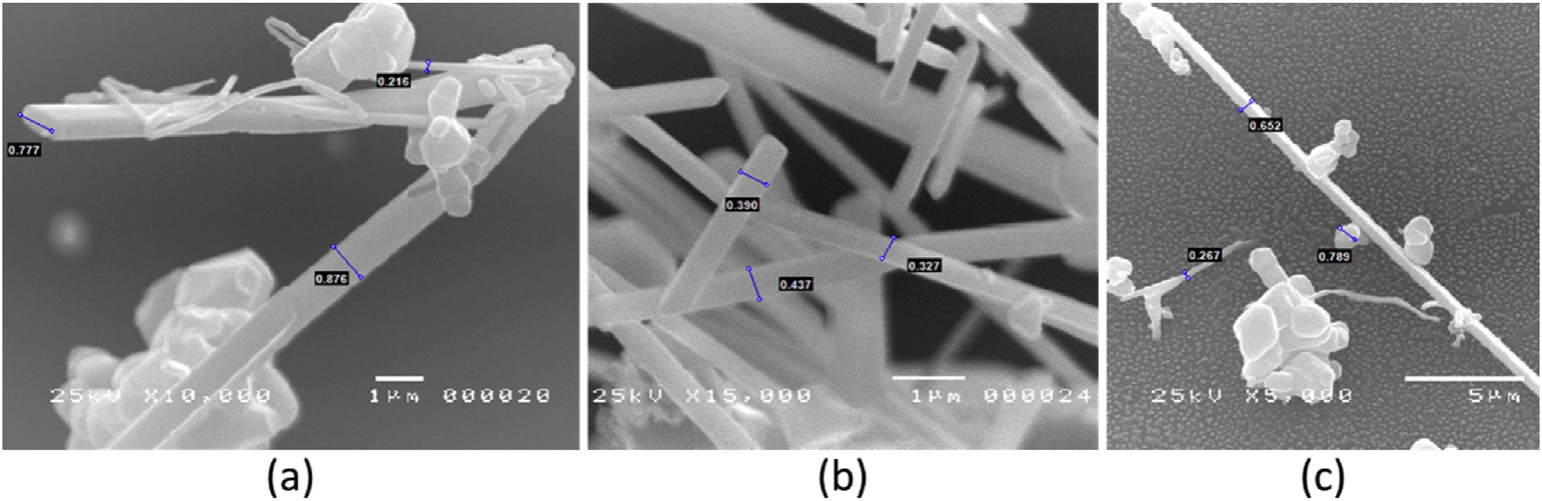
Synthesis Ag/TiO_2_ nanocomposite materials using temperature and baking time (1000 °C, 8 hours) as a parameter with three different zones of measurement. (a) zone 1, (b) zone 2, (c) zone 3.

**Fig. 8:**
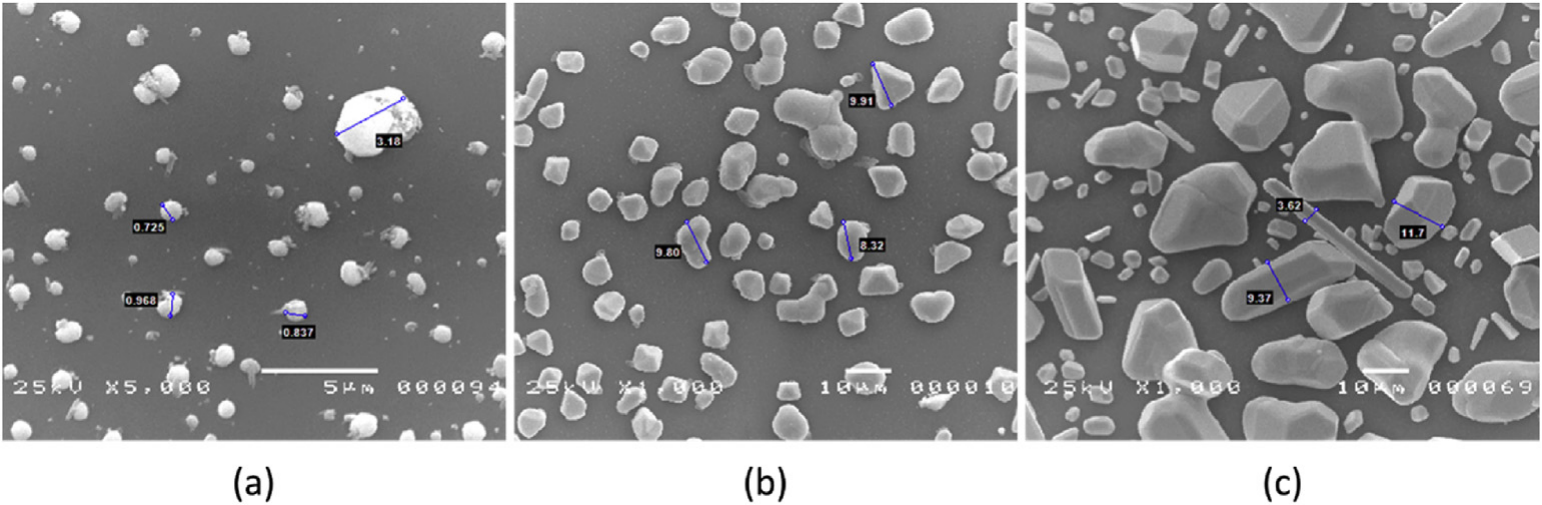
SEM image of Ag/TiO_2_ nanocomposite material with different baking time. (a) 1200 °C, 4 hours, (b) 1200 °C, 6 hours, and (c) 1200 °C, 8 hours.

Specimens measurement technique in different parameter is shown in Fig. 7 and Fig. 8. Different shape of nanocomposite material in the different zone is shown in Fig. 7. It is shown that different shape also has different measurable. Fig. 8 shows the different shape of nanocomposite material in different baking time. The measurement value shows that diameter of the material shows different with the increase of baking time.
