# Supplementary material for: Dataset of material measurement based on SEM images of Ag/TiO2 nanocomposite material synthesized via Horizontal Vapor Phase Growth (HVPG) technique
Source: Data Brief. 2020 Jan 3;28:105018. doi: 10.1016/j.dib.2019.105018 (PMC6950646; doi:10.1016/j.dib.2019.105018)
Supplement: Multimedia component 1 [file mmc1.pptx]

## Slide 1
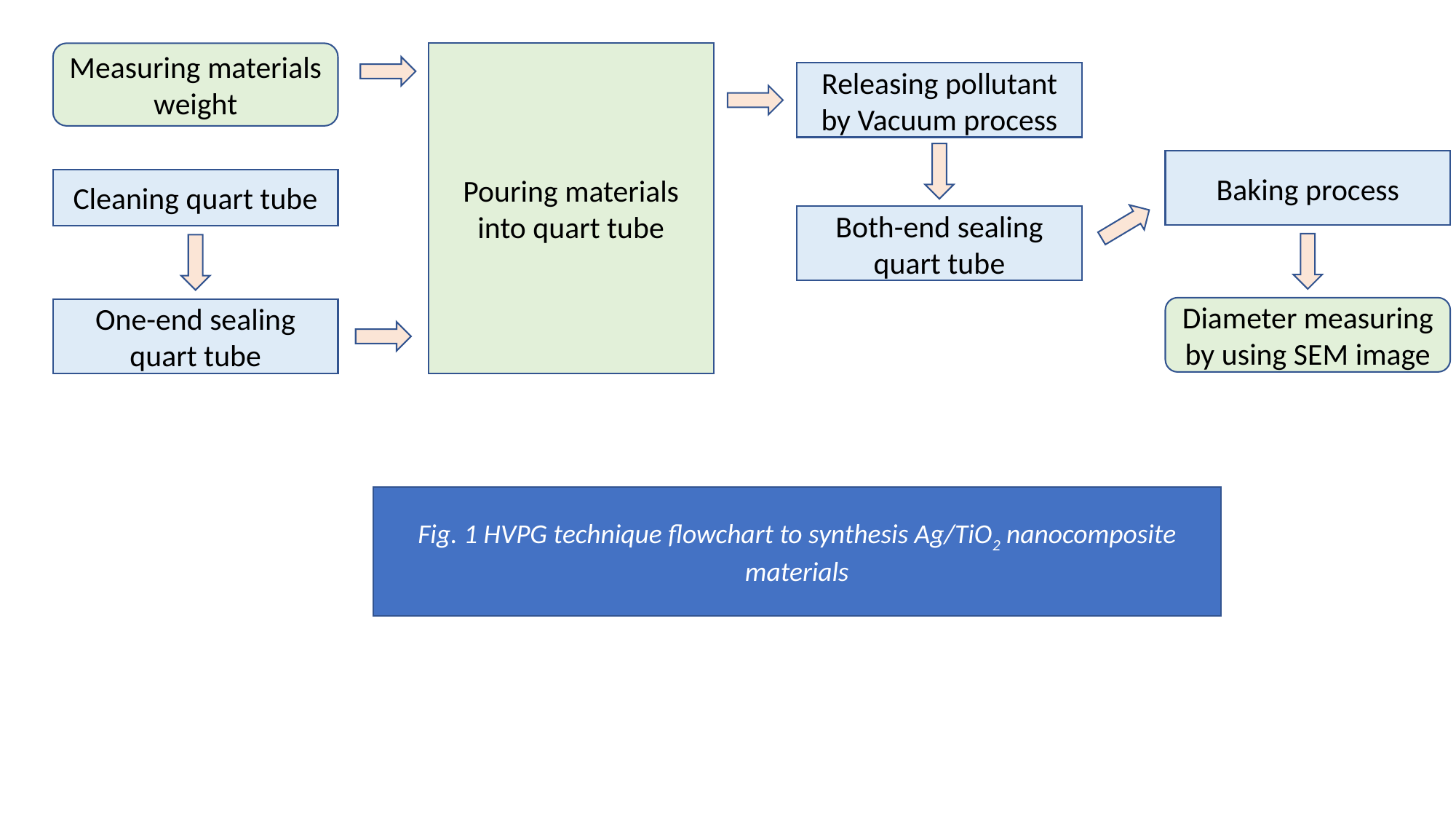

Measuring materials weight
Pouring materials into quart tube
Releasing pollutant by Vacuum process
Baking process
Cleaning quart tube
Both-end sealing quart tube
Diameter measuring by using SEM image
One-end sealing quart tube
Fig. 1 HVPG technique flowchart to synthesis Ag/TiO2 nanocomposite materials

## Slide 2
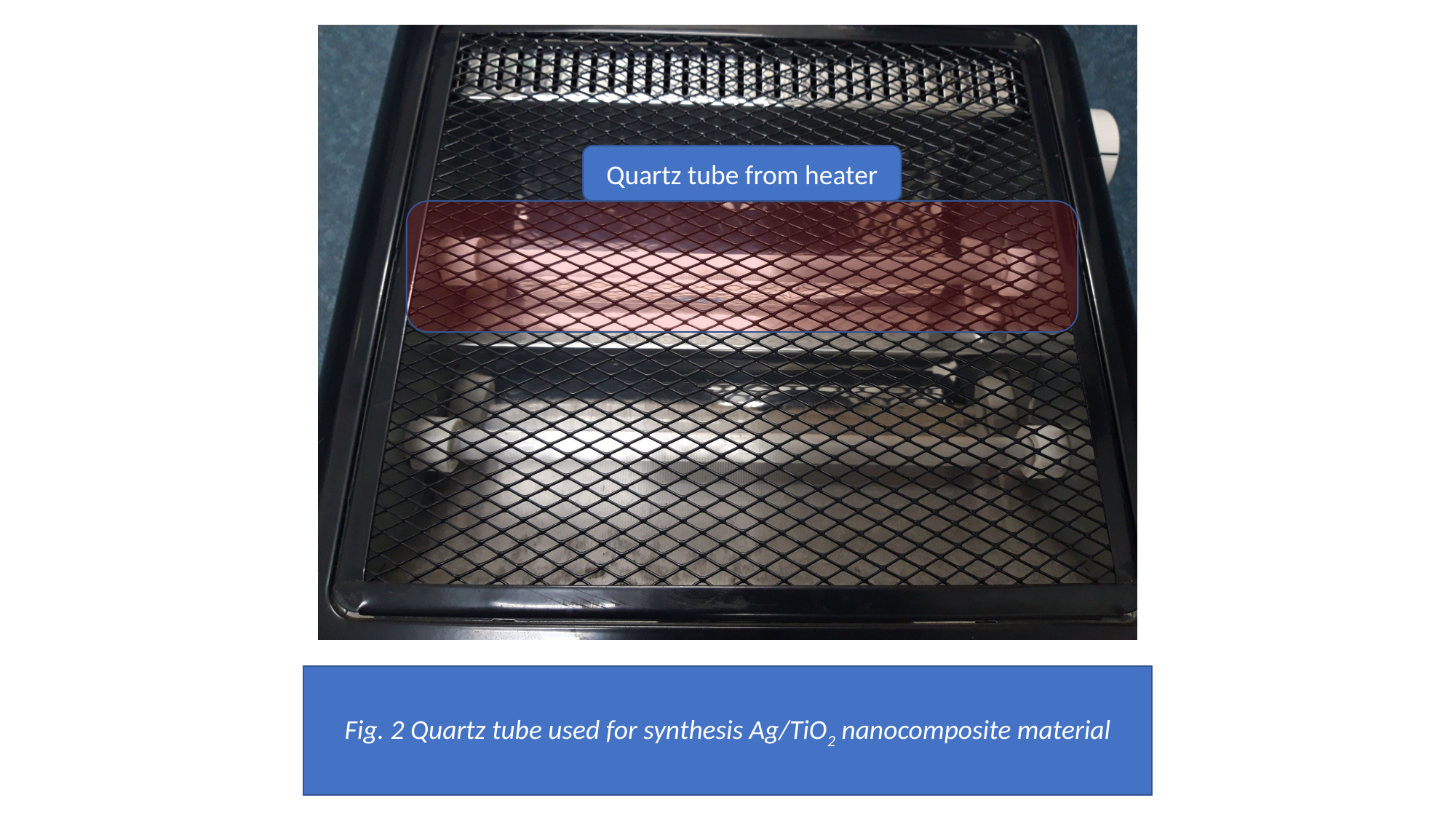

Quartz tube from heater
Fig. 2 Quartz tube used for synthesis Ag/TiO2 nanocomposite material

## Slide 3
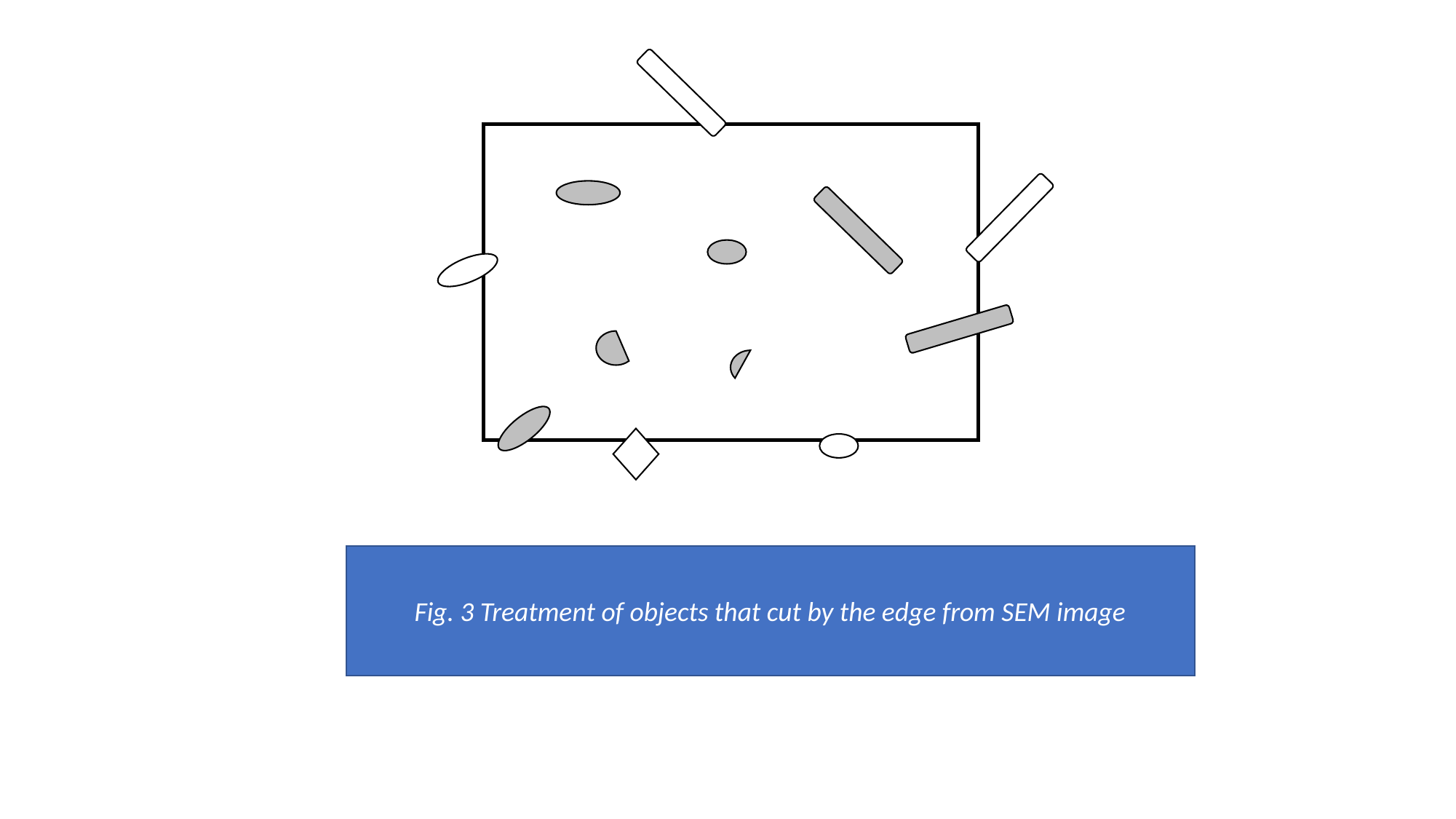

Fig. 3 Treatment of objects that cut by the edge from SEM image

## Slide 4
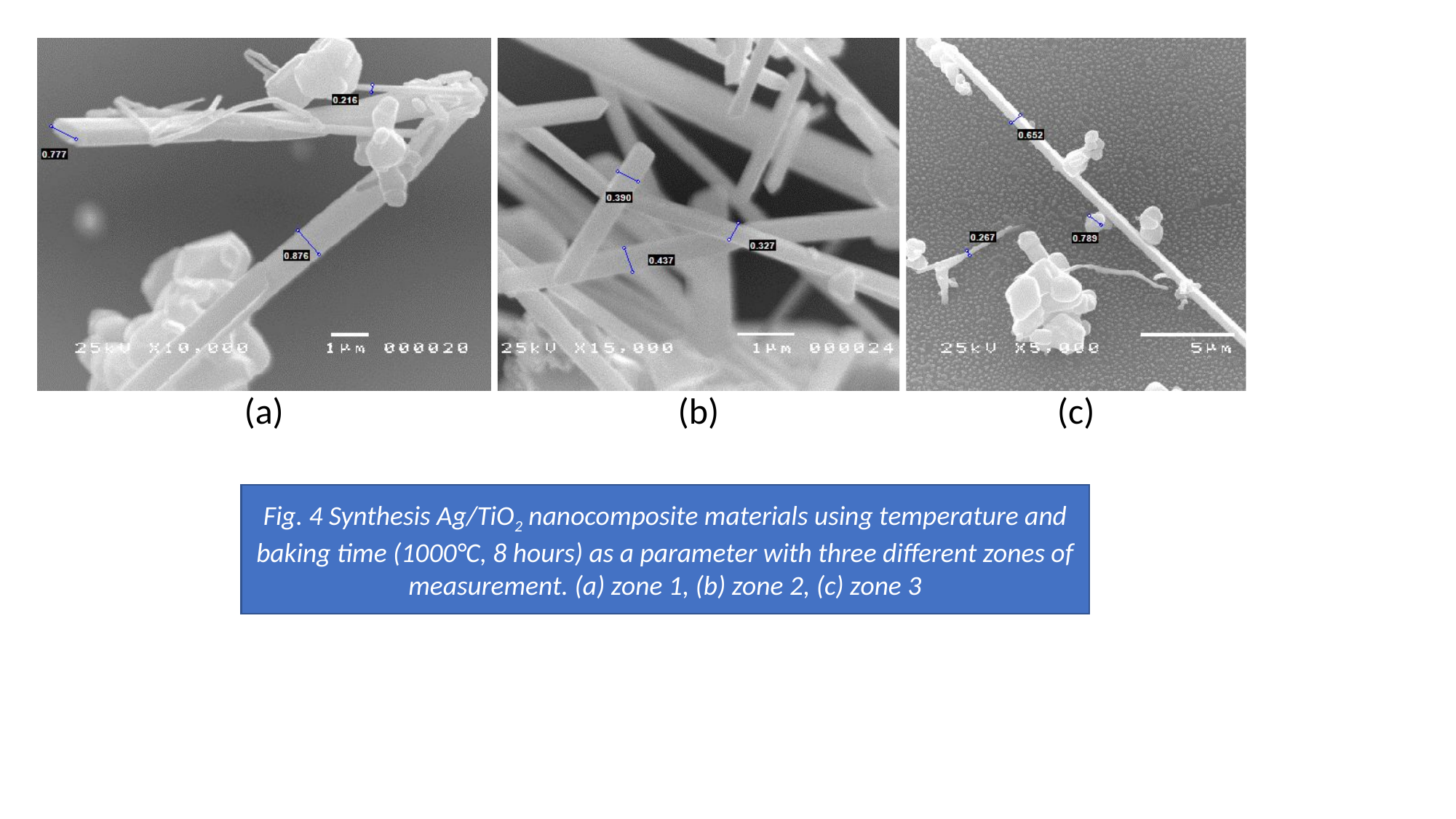

(a)
(b)
(c)
Fig. 4 Synthesis Ag/TiO2 nanocomposite materials using temperature and baking time (1000°C, 8 hours) as a parameter with three different zones of measurement. (a) zone 1, (b) zone 2, (c) zone 3

## Slide 5
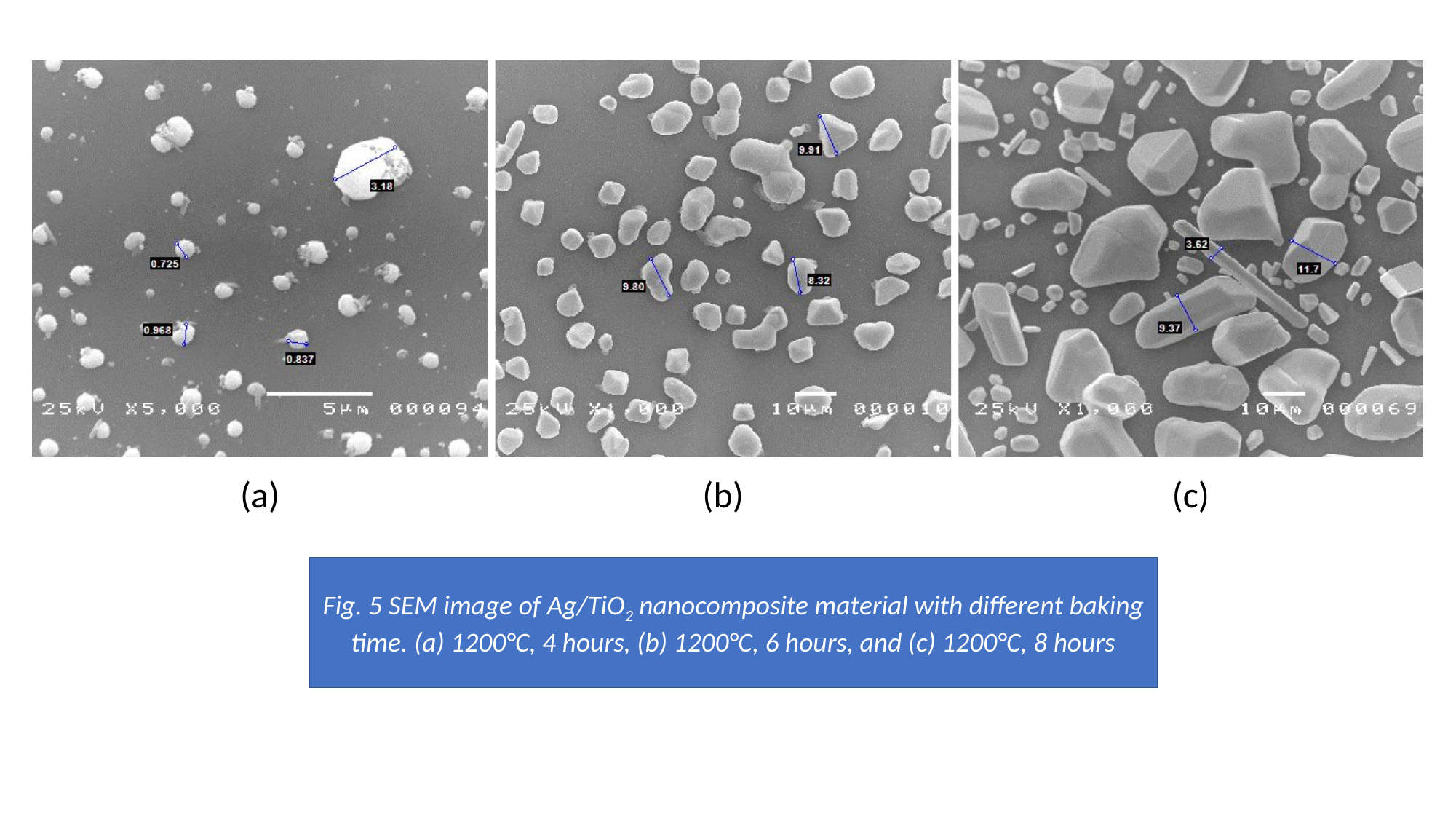

(a)
(b)
(c)
Fig. 5 SEM image of Ag/TiO2 nanocomposite material with different baking time. (a) 1200°C, 4 hours, (b) 1200°C, 6 hours, and (c) 1200°C, 8 hours
