# Supplementary figures and images for: Dataset of material measurement based on SEM images of Ag/TiO2 nanocomposite material synthesized via Horizontal Vapor Phase Growth (HVPG) technique
Source: Data Brief. 2020 Jan 3;28:105018. doi: 10.1016/j.dib.2019.105018 (PMC6950646; doi:10.1016/j.dib.2019.105018)

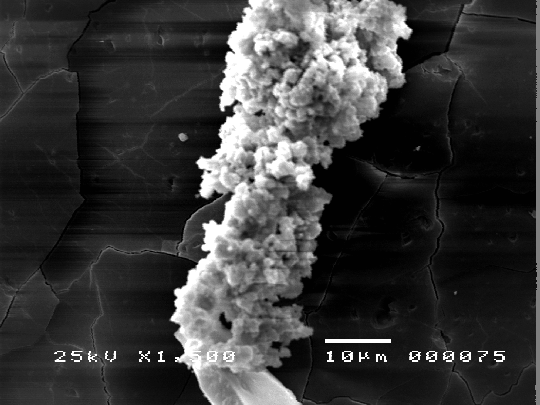

Supplement: Multimedia component 3 [file mmc3.zip › SEM image data/1000 4hTube 2/Measurement tube 2/1a.BMP]

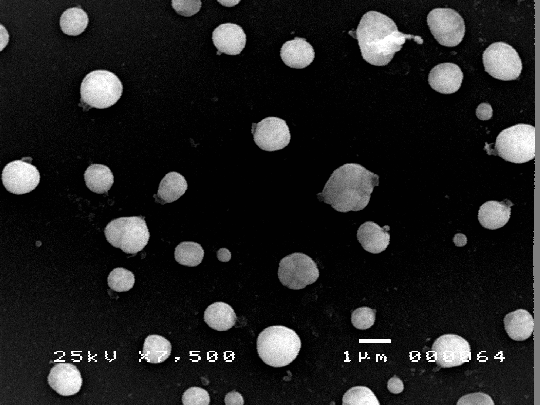

Supplement: Multimedia component 3 [file mmc3.zip › SEM image data/1000 4hTube 2/Measurement tube 2/2b.BMP]

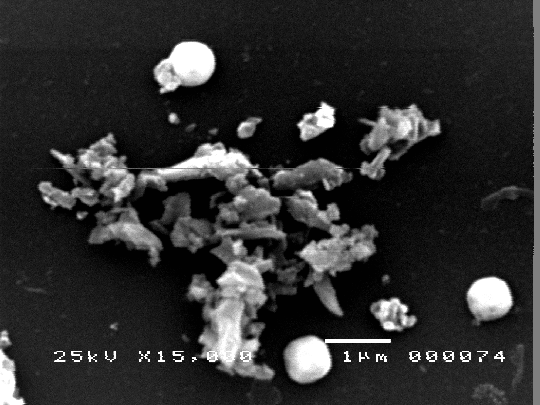

Supplement: Multimedia component 3 [file mmc3.zip › SEM image data/1000 4hTube 2/Measurement tube 2/3c.BMP]

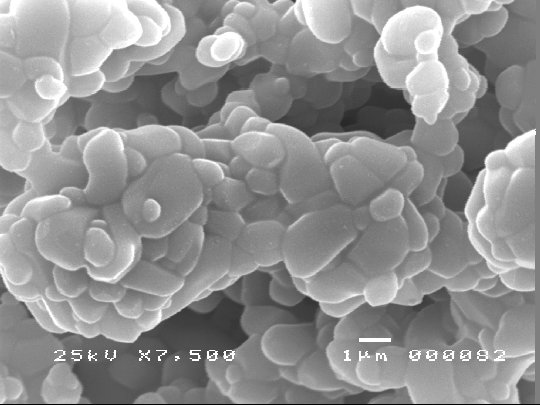

Supplement: Multimedia component 3 [file mmc3.zip › SEM image data/1000 6hTube 4/Measurement/1a.BMP]

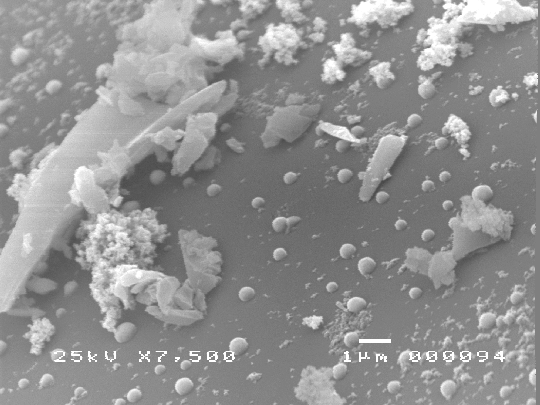

Supplement: Multimedia component 3 [file mmc3.zip › SEM image data/1000 6hTube 4/Measurement/2b.BMP]

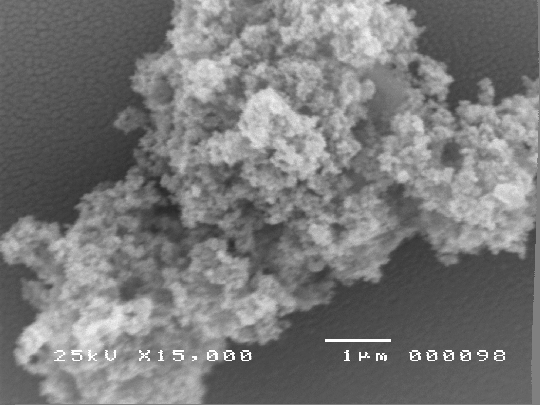

Supplement: Multimedia component 3 [file mmc3.zip › SEM image data/1000 6hTube 4/Measurement/3c.BMP]

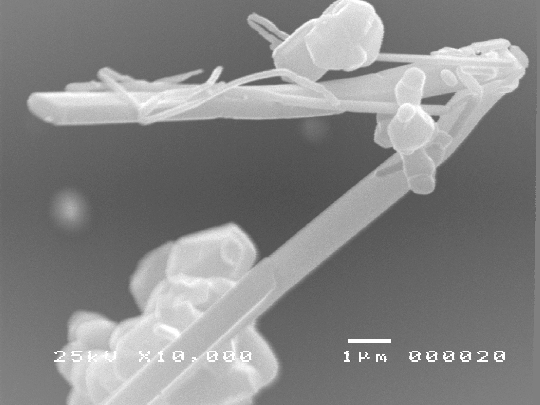

Supplement: Multimedia component 3 [file mmc3.zip › SEM image data/1000 8hTube 6/Measurement/1a.BMP]

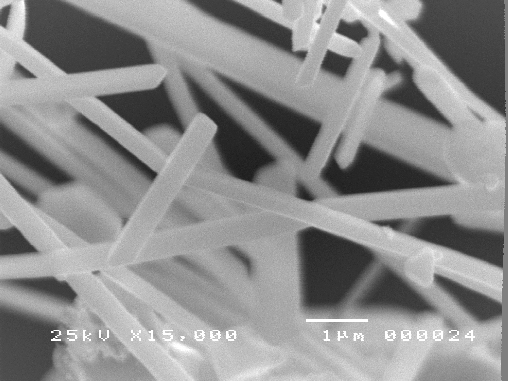

Supplement: Multimedia component 3 [file mmc3.zip › SEM image data/1000 8hTube 6/Measurement/2b.BMP]

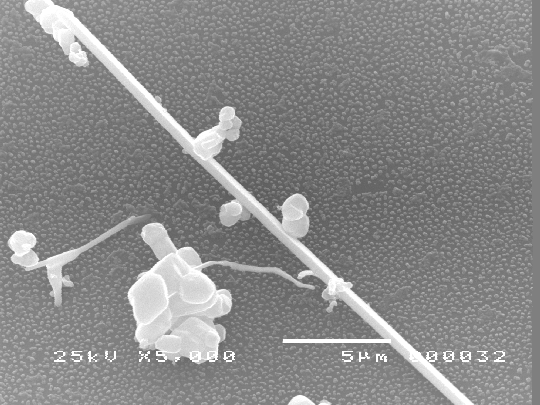

Supplement: Multimedia component 3 [file mmc3.zip › SEM image data/1000 8hTube 6/Measurement/3c.BMP]

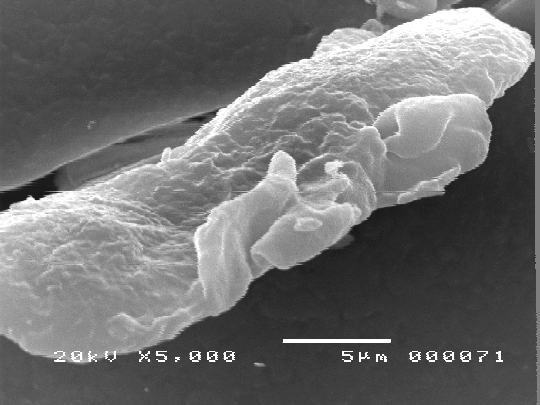

Supplement: Multimedia component 3 [file mmc3.zip › SEM image data/1200 4hTube 7/Measurement/1a.BMP]

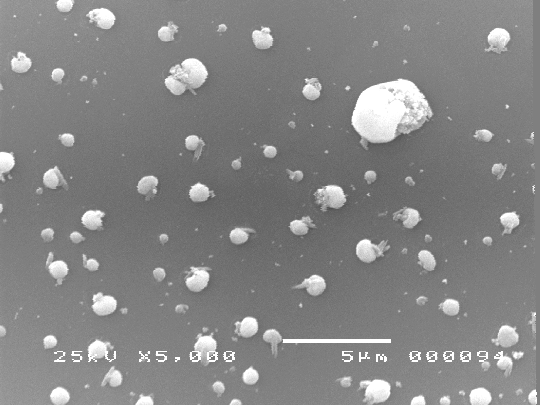

Supplement: Multimedia component 3 [file mmc3.zip › SEM image data/1200 4hTube 7/Measurement/2b.BMP]

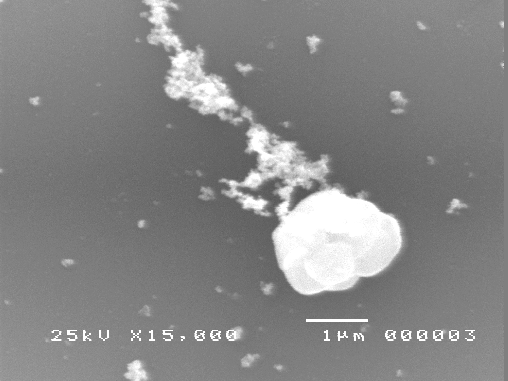

Supplement: Multimedia component 3 [file mmc3.zip › SEM image data/1200 4hTube 7/Measurement/3c.BMP]

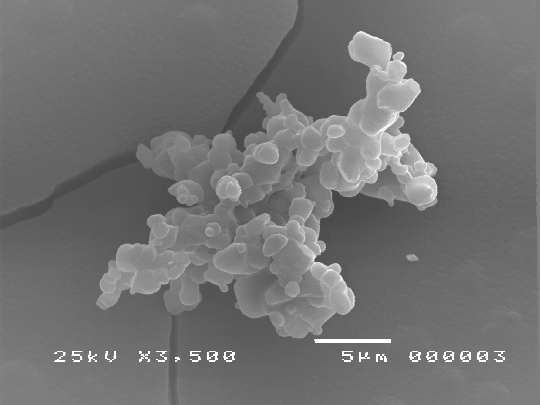

Supplement: Multimedia component 3 [file mmc3.zip › SEM image data/1200 6hTube 8/Measurement/1a.BMP]

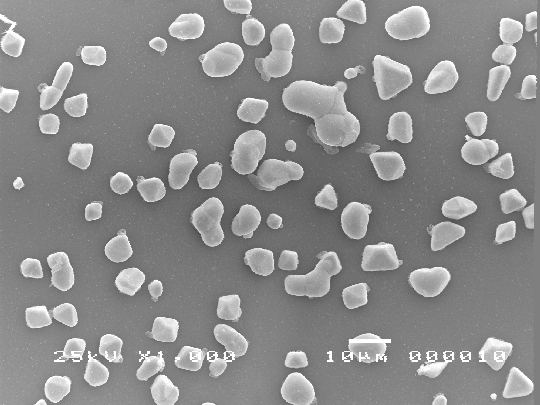

Supplement: Multimedia component 3 [file mmc3.zip › SEM image data/1200 6hTube 8/Measurement/2b.BMP]

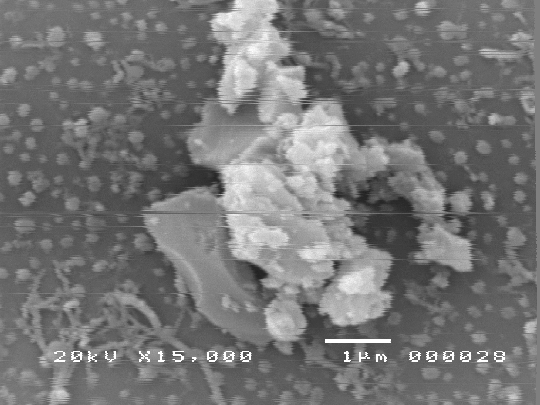

Supplement: Multimedia component 3 [file mmc3.zip › SEM image data/1200 6hTube 8/Measurement/3c.BMP]

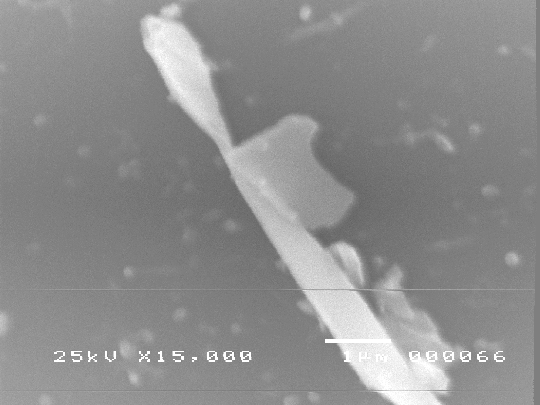

Supplement: Multimedia component 3 [file mmc3.zip › SEM image data/1200 8hTube 9/Measurement/1a.BMP]

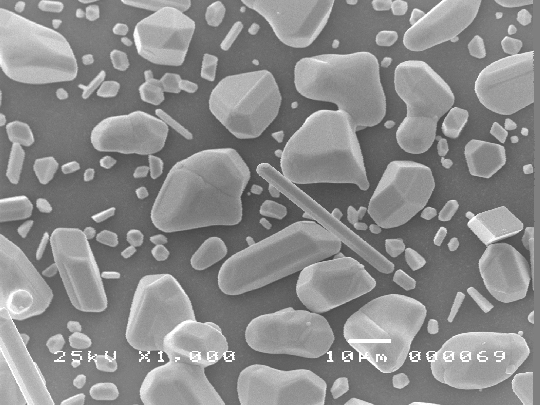

Supplement: Multimedia component 3 [file mmc3.zip › SEM image data/1200 8hTube 9/Measurement/2b.BMP]

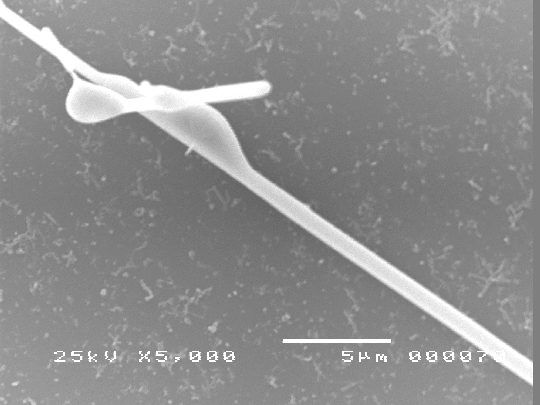

Supplement: Multimedia component 3 [file mmc3.zip › SEM image data/1200 8hTube 9/Measurement/3c.BMP]

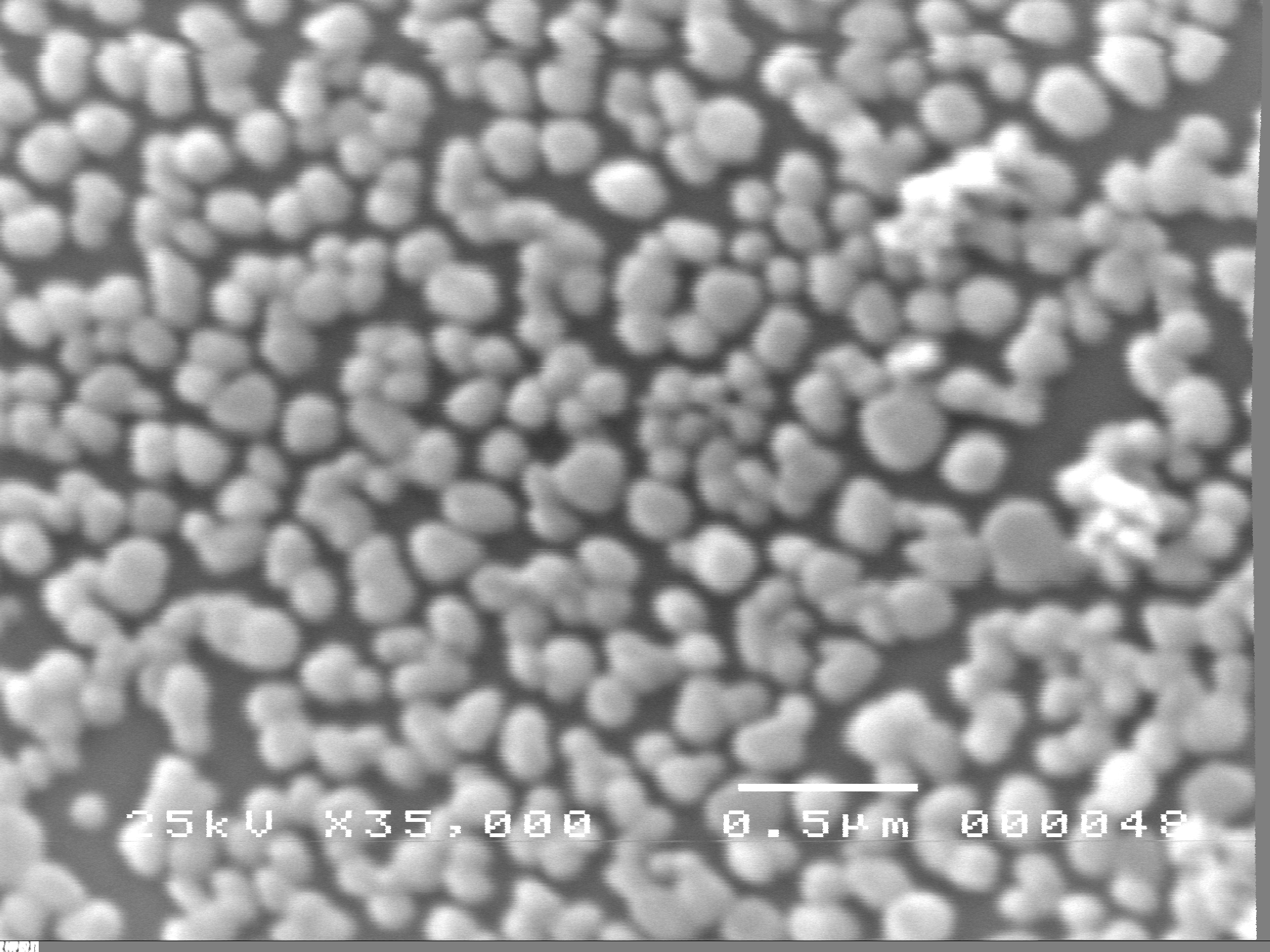

Supplement: Multimedia component 3 [file mmc3.zip › SEM image data/800 4hTube 1/measurement tube 1/1a.BMP]

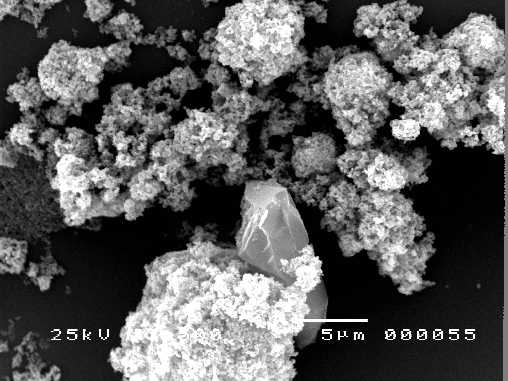

Supplement: Multimedia component 3 [file mmc3.zip › SEM image data/800 4hTube 1/measurement tube 1/2b.BMP]

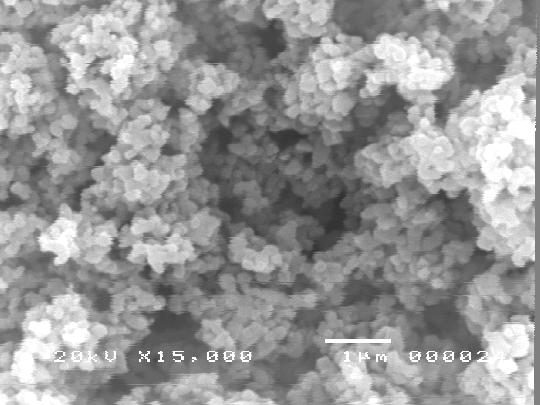

Supplement: Multimedia component 3 [file mmc3.zip › SEM image data/800 4hTube 1/measurement tube 1/3c.BMP]

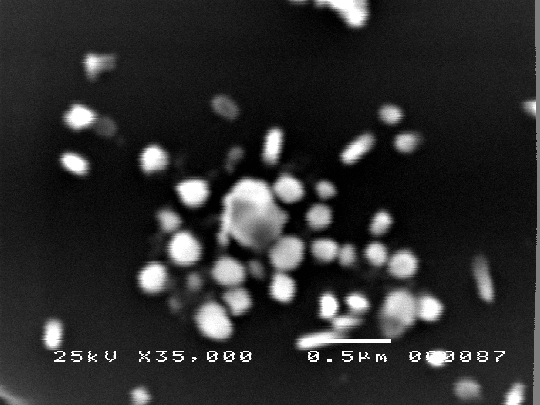

Supplement: Multimedia component 3 [file mmc3.zip › SEM image data/800 6hTube 3/Measurement/1a.BMP]

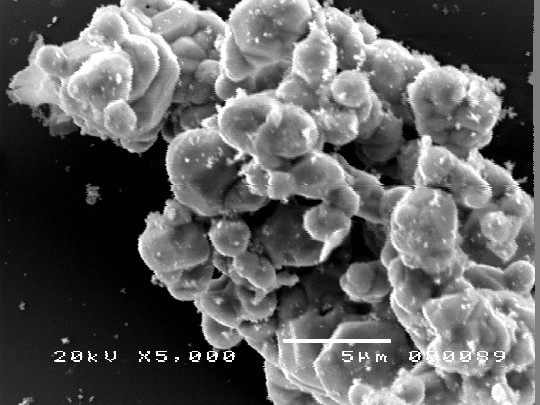

Supplement: Multimedia component 3 [file mmc3.zip › SEM image data/800 6hTube 3/Measurement/2b.BMP]

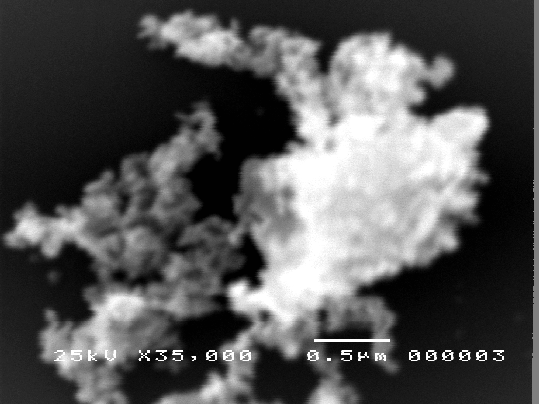

Supplement: Multimedia component 3 [file mmc3.zip › SEM image data/800 6hTube 3/Measurement/3c.BMP]

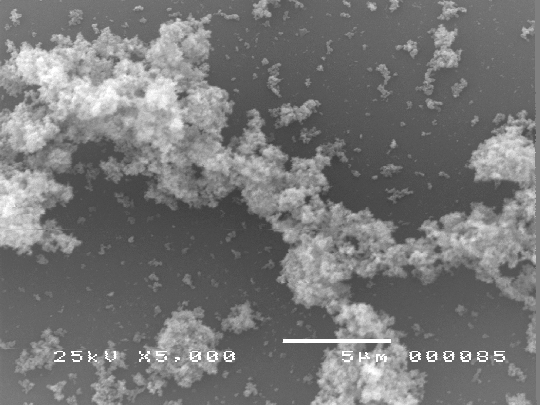

Supplement: Multimedia component 3 [file mmc3.zip › SEM image data/800 8hTube 5/Measurement/1a.BMP]

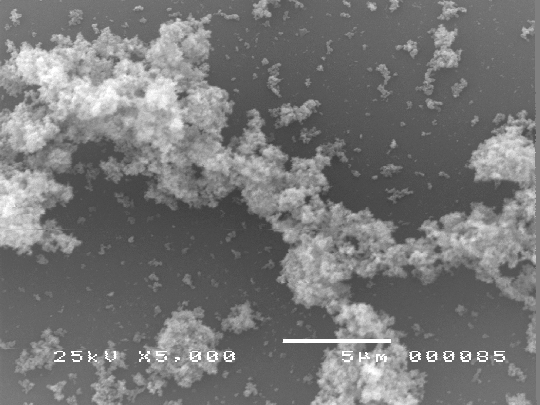

Supplement: Multimedia component 3 [file mmc3.zip › SEM image data/800 8hTube 5/Measurement/1b.BMP]

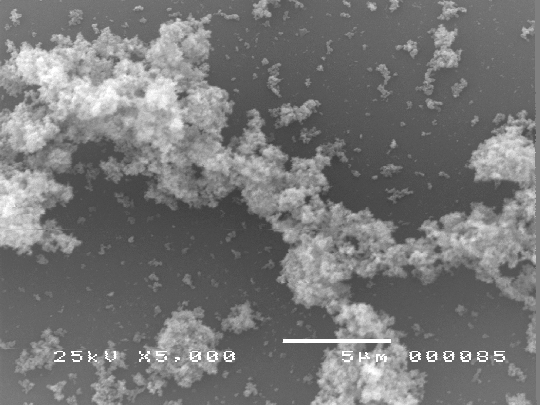

Supplement: Multimedia component 3 [file mmc3.zip › SEM image data/800 8hTube 5/Measurement/1c.BMP]

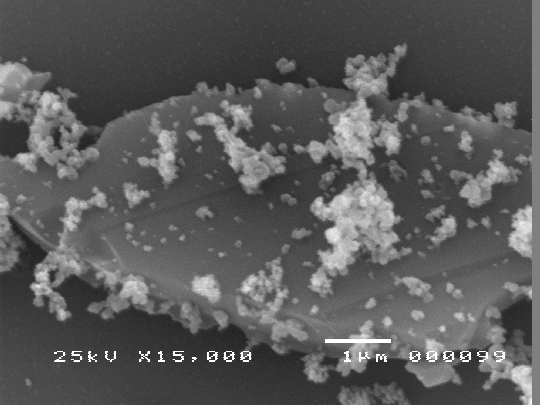

Supplement: Multimedia component 3 [file mmc3.zip › SEM image data/800 8hTube 5/Measurement/2a.BMP]

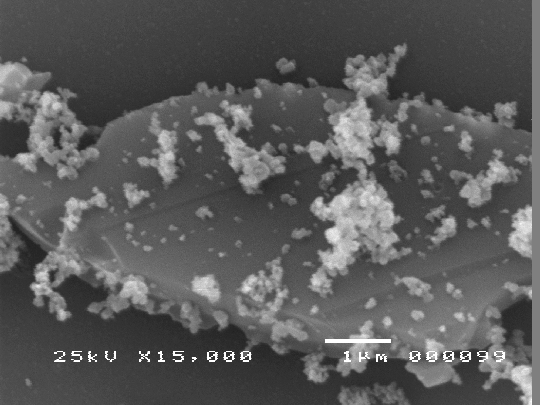

Supplement: Multimedia component 3 [file mmc3.zip › SEM image data/800 8hTube 5/Measurement/2b.BMP]

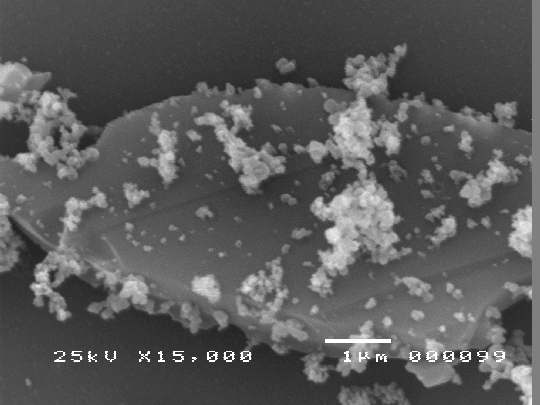

Supplement: Multimedia component 3 [file mmc3.zip › SEM image data/800 8hTube 5/Measurement/2c.BMP]

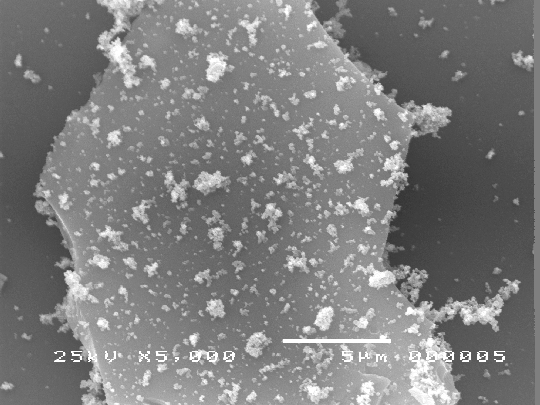

Supplement: Multimedia component 3 [file mmc3.zip › SEM image data/800 8hTube 5/Measurement/3a.BMP]

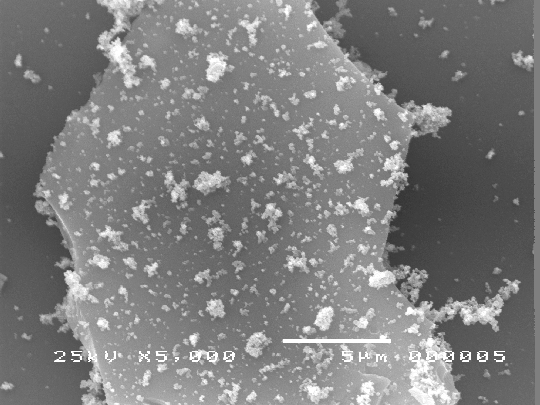

Supplement: Multimedia component 3 [file mmc3.zip › SEM image data/800 8hTube 5/Measurement/3b.BMP]

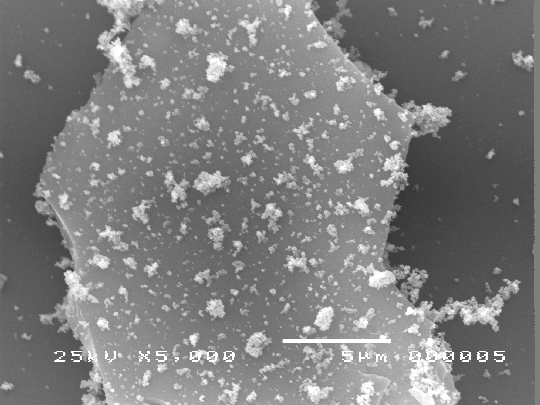

Supplement: Multimedia component 3 [file mmc3.zip › SEM image data/800 8hTube 5/Measurement/3c.BMP]

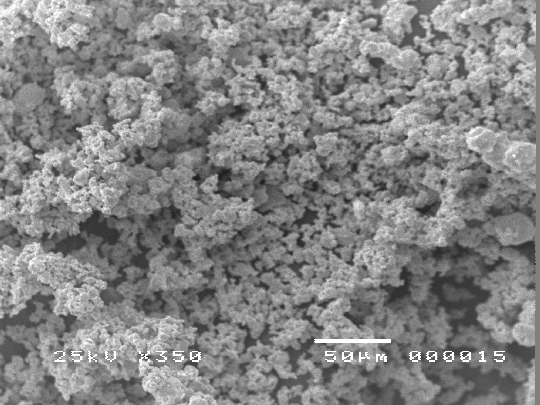

Supplement: Multimedia component 3 [file mmc3.zip › SEM image data/Silver Powder/Measurement silver/1a.BMP]

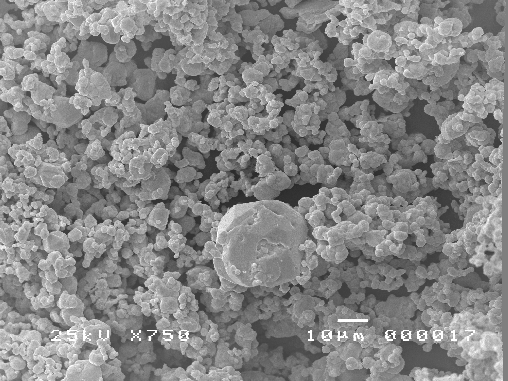

Supplement: Multimedia component 3 [file mmc3.zip › SEM image data/Silver Powder/Measurement silver/2b.BMP]

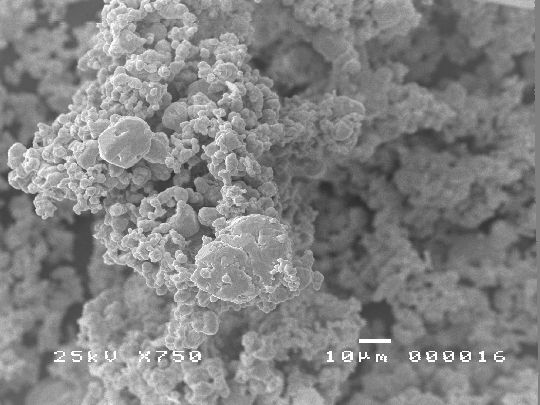

Supplement: Multimedia component 3 [file mmc3.zip › SEM image data/Silver Powder/Measurement silver/3c.BMP]

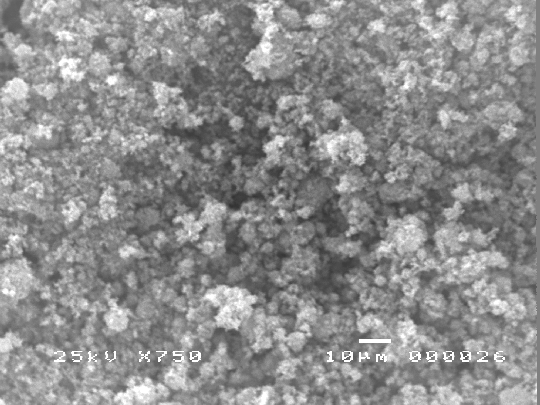

Supplement: Multimedia component 3 [file mmc3.zip › SEM image data/Titanium Dioxide Powder/Measurement titanium/1a.BMP]

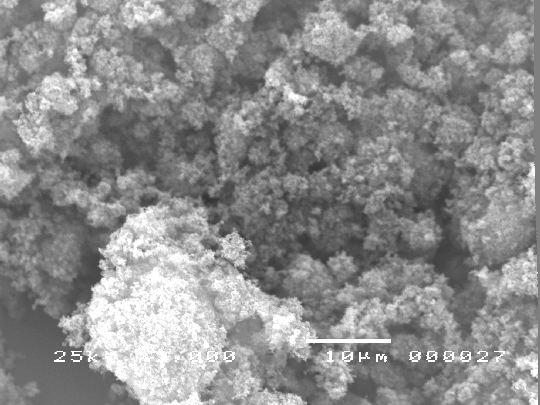

Supplement: Multimedia component 3 [file mmc3.zip › SEM image data/Titanium Dioxide Powder/Measurement titanium/2b.BMP]

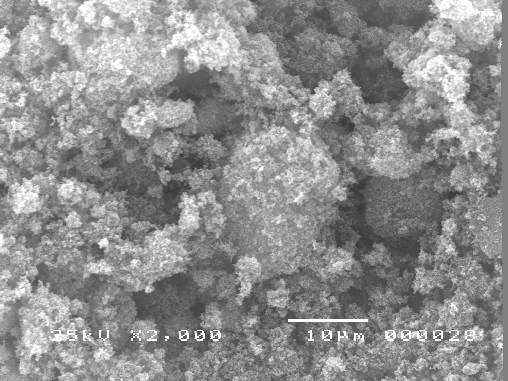

Supplement: Multimedia component 3 [file mmc3.zip › SEM image data/Titanium Dioxide Powder/Measurement titanium/3c.BMP]
